# Grammatical Language Impairment in Autism Spectrum Disorder: Exploring Language Phenotypes Beyond Standardized Testing

**DOI:** 10.3389/fpsyg.2017.00532

**Published:** 2017-04-18

**Authors:** Kacie Wittke, Ann M. Mastergeorge, Sally Ozonoff, Sally J. Rogers, Letitia R. Naigles

**Affiliations:** ^1^Department of Speech, Language, and Hearing Sciences, University of Connecticut, StorrsCT, USA; ^2^Human Development and Family Studies, Texas Tech University, LubbockTX, USA; ^3^UC Davis MIND Institute, SacramentoCA, USA; ^4^Department of Psychology, University of Connecticut, StorrsCT, USA

**Keywords:** autism spectrum disorder, language impairment, grammar, language samples

## Abstract

Linguistic and cognitive abilities manifest huge heterogeneity in children with autism spectrum disorder (ASD). Some children present with commensurate language and cognitive abilities, while others show more variable patterns of development. Using spontaneous language samples, we investigate the presence and extent of grammatical language impairment in a heterogeneous sample of children with ASD. Findings from our sample suggest that children with ASD can be categorized into three meaningful subgroups: those with normal language, those with marked difficulty in grammatical production but relatively intact vocabulary, and those with more globally low language abilities. These findings support the use of sensitive assessment measures to evaluate language in autism, as well as the utility of within-disorder comparisons, in order to comprehensively define the various cognitive and linguistic phenotypes in this heterogeneous disorder.

## Introduction

According to the *Diagnostic and Statistical Manual of Mental Disorders, Fifth Edition* (DSM-5), the criteria for a diagnosis of autism spectrum disorder (ASD) include persistent deficits in social communication and interaction, as well as restricted and repetitive behaviors or interests (American Psychiatric Association, 2013). Children who meet criteria for ASD may have an accompanying language impairment, but this is not required for making the diagnosis. As ASD exists on a continuum, there is significant heterogeneity in the phenotypic presentation of individuals with this disorder, ranging from mild to more severe impairments. This range of abilities is also seen in the language skills of children with ASD: some present with intact language skills, while others develop little or no language (Tager-Flusberg, 2004). Moreover, within the set of children who do acquire language, pragmatic skills have been found to be consistently poor whereas grammatical abilities can vary widely, even in high-functioning individuals with autism. Some children present with grammar in the average range (Kjelgaard and Tager-Flusberg, 2001; Tek et al., 2014), while others have notable difficulties with grammar (Roberts et al., 2004; Eigsti et al., 2007; Tek et al., 2014; Durrleman and Delage, 2016; Modyanova et al., 2017). Several researchers have even suggested that a subset of children with ASD meet criteria for a co-morbid specific language impairment (SLI), as they appear to have primary difficulties with grammar despite normal cognitive abilities. However, this proposition has continued to be controversial (e.g., Williams et al., 2008; Riches et al., 2010; Tuller et al., 2017), and requires extensive language testing from both populations to be established. Our goals in this paper focus instead on fleshing out the language heterogeneity *within* the ASD population; in particular, discovering the extent to which meaningful linguistic subgroups emerge when grammatical usage is scrutinized in detail. Also novel to our investigation is the borrowing of spontaneous language measures from the SLI literature for lexical and grammatical profiling, and the inclusion of a relatively large (*n* = 82) sample of 5-year old children with ASD.

### Language in Autism: a Focus on Grammar

Tager-Flusberg et al. (2005) review the range of linguistic abilities in children across the autism spectrum, making two major distinctions. First, some children with ASD fail to acquire spoken language skills beyond a basic or minimal level, which may range from no spoken words to fewer than 20–30 words (Kasari et al., 2013); about 30% of children with autism fall into this group (Tager-Flusberg and Kasari, 2013). Second, within the group of children who are verbal, some present with normal language while others have a notable language deficit, including difficulties with the understanding and use of grammar (Tager-Flusberg and Joseph, 2003; Norbury, 2017). In the literature, these latter two groups are often distinguished with the terminology autism language normal (ALN) and autism with language impairment (ALI).

A number of grammatical areas have been found to be problematic when children with ALI are compared to typically developing (TD) peers. When probed during an elicited production task, children with ALI produce significantly fewer markers of past tense -*ed* and third person singular -*s* (Roberts et al., 2004). Similar tense and agreement omissions have been documented in other studies using spontaneous language samples (Bartolucci et al., 1980; Tager-Flusberg, 1989). Eigsti et al. (2007) found that 5-year-old children with ASD, who were matched to younger TD children on vocabulary and non-verbal IQ, exhibited considerably less complex language than the younger TD group, producing fewer past tense markers as well as fewer Wh-questions. Grammatical errors are also seen in pronoun use. While pronoun reversals (e.g., “you” for “I”) are much less prominent than once thought, they are produced more frequently by preschoolers with ASD than TD peers (Naigles et al., 2016). Distinguishing personal and reflexive pronouns has also been found to be challenging for children with ALI (Perovic et al., 2013); moreover, French-speaking children with ALI demonstrate notable difficulty with pronominal clitics (Durrleman and Delage, 2016; Tuller et al., 2017). Findings such as these suggest that grammatical challenges involving language production do arise in ASD; however, there are a number of unresolved questions. First, to what extent is this grammatical impairment independent of the child’s non-verbal IQ and/or vocabulary level? While some baseline level of non-verbal IQ seems required for children to achieve phrase speech at all (Anderson et al., 2007; Wodka et al., 2013; Tek et al., 2014), the demonstration that impaired grammatical knowledge exists in children whose non-verbal cognition is within normal limits suggests that the acquisition of grammar depends, at least somewhat, on factors external to general cognition (e.g., Lewis and Landau, 2015; Valian, 2015). This is seen most notably in SLI, but it is less well defined in ASD, which leads to the second question: how pervasively across the autism spectrum do these grammatical impairments arise, and do the same types of impairments recur?

Addressing the first question naturally leads to another population of children with language disorders; namely, those with SLI. Definitionally, children with SLI present with language impairment despite having non-verbal IQ within the normal range and no other developmental or neurological disorder (Leonard, 2014). While the definition of “normal range” cognition varies between SLI researchers, with some considering it synonymous with average range performance and others considering it as scoring above the intellectual disability range (see Gallinat and Spaulding, 2014 for review), the language deficits in SLI are more well-defined. Hallmark language characteristics associated with SLI include particular difficulty with grammatical morphology, such as tense and agreement markers (Rice et al., 1995), as well as pronoun errors when marking case, gender, and number (Van der Lely and Stollwerck, 1997; Moore, 2001). Although research by Sheng and McGregor (2010), McGregor et al. (2013) has found that children with SLI show qualitative differences in their vocabulary knowledge and speed of word learning, overall, children with SLI perform well on tests of vocabulary (e.g., Spaulding et al., 2013). Thus, morphosyntactic errors are typically most notable whereas vocabulary is a relative strength. In the current paper, we borrow some measures from SLI research to further investigate the heterogeneity of grammatical impairment in ASD.

Research addressing the second question has primarily attempted to subgroup children with ASD based on their language abilities. However, not all research in this domain has focused specifically on grammatical language abilities; rather, language has been explored more broadly. For example, Kjelgaard and Tager-Flusberg (2001) found that several distinct language phenotypes of autism emerged from their sample of 89 children. Assessing performance on a variety of standardized language measures, they found three language subgroups. Children in their *normal* and *impaired* language groups had commensurately high and low non-verbal and language abilities, respectively. Yet, children in the *borderline* group reportedly resembled children with SLI, as they had normal non-verbal IQ scores but language below average (Kjelgaard and Tager-Flusberg, 2001). Their findings were limited to certain aspects of language, as the majority of testing focused on vocabulary and none of the measures they used contained detailed indices of grammar. Tager-Flusberg and Joseph (2003) reported similar findings from two samples of school-age children. The first sample showed that children with borderline and impaired language manifested grammatical impairments disproportionately more severe than lexical ones, whereas their second sample showed that some children with ASD have verbal scores lower than non-verbal ones. However, no detailed grammatical measures were provided for those participants either. Other researchers have attempted to subgroup children with ASD based on language abilities (e.g., Anderson et al., 2007; Rapin et al., 2009), but until recently the measures were drawn from standardized tests, which did not enable detailed analysis of grammatical abilities (for more recent research see Durrleman and Delage, 2016; Modyanova et al., 2017; Tuller et al., 2017). Thus, research to date suggests that there may be multiple subgroups within the category of ALI, as demonstrated by research from Kjelgaard and Tager-Flusberg (2001), Tager-Flusberg and Joseph (2003), but it is unclear how lexical and grammatical abilities might differ among those subgroups.

In sum, research using standardized testing has shown that some children with ASD whose language scores—probably also including grammar—are on par with their TD age-mates; hence, with both language and non-verbal cognitive abilities high/intact, they are referred to as ALN. Researchers and clinicians agree, as well, on the existence of children with ASD whose language levels are minimal to null, and whose cognitive scores are correspondingly low—those who are minimally or non-verbal (NV). What is not yet clear are the characteristics of the children whose abilities range in between these two ends of the spectrum. Research has shown that these children present with weaknesses in their grammatical production skills; however, there may yet be different subgroups within this range. We suspect there may be at least two different subgroups in ALI, including those with normal non-verbal IQ but impaired language, as well as those whose language *and* cognitive scores are below their age level. How prevalent these groups might be, and to what extent their grammatical and lexical production is similar to and different from each other is poorly defined. It is also unclear how these groups compare to those with ALN on measures of both lexical and grammatical development.

No research thus far has compared these possible subgroups on grammar in any detail, especially because of the reliance on standardized language testing in past studies. As research on SLI has demonstrated, standardized tests are not necessarily sensitive to the types of grammatical deficits typically seen in children with SLI (Greenslade et al., 2009); thus, they may also not be sensitive to grammatical deficits in ASD, especially for examining possible subgroups within ALI. Spontaneous language samples, a methodology that is particularly sensitive to the expressive language deficits in SLI (e.g., Hewitt et al., 2005; Rice et al., 2010), could be an ideal way to capture the range of grammatical abilities in ASD.

### Spontaneous Language Samples: Examining Heterogeneity of Grammar in Children with Language Impairment

While most research to date exploring SLI in ASD has focused on comparing these two disorders, our focus is on how the literature in SLI can provide guidance for how to examine grammar in ASD. In SLI, spontaneous language samples have been used to examine features of their language, many of which have illuminated how children with SLI have many notable differences in grammatical production skills relative to their TD peers. We propose that these SLI-relevant language variables, as described below, should be considered in exploring the grammatical characteristics of language subgroups in ASD.

Children with SLI have been found to produce more grammatical errors overall starting at a young age. For example, Eisenberg and Guo (2013) calculated the frequency of grammatical vs. ungrammatical utterances in a sample of 3-year-old children with SLI and found that, on average, 62% of their utterances were ungrammatical. This is in contrast to their TD peers, only 29% of whose utterances were ungrammatical (Eisenberg et al., 2012). Dunn et al. (1996) also found group differences in total grammatical errors between 4-year-old children with SLI relative to their TD peers; the mean percentage of ungrammatical language in the SLI group was 23.56% of total utterances compared to 10.97% in the TD group. While there are no norms for children’s percentage of grammatical errors across language development, nor is there currently a clinically meaningful cut-off for frequency of grammatical errors in a clinical population like SLI, these studies demonstrate that children with SLI produce far more ungrammatical utterances than their peers. One notable observation from these two studies is that while errors become less frequent across both groups from ages 3 to 4, four-year-old children with SLI (Dunn et al., 1996) seem to produce errors at frequency rates similar to TD 3-year olds (Eisenberg et al., 2012). While cross-study comparisons should be made cautiously given the small sample sizes of these studies, it could be predicted that 5-year-old children with SLI might have grammatical errors at a frequency rate similar to TD 4-year-olds, i.e., somewhere around 10%.

Examination of grammar in SLI has also shown that there are specific markers that are particularly sensitive diagnostic indicators of language impairment, particularly in the preschool and Kindergarten years (e.g., Rice et al., 1995; Eisenberg and Guo, 2013). For instance, Bedore and Leonard (1998) analyzed language samples from both SLI and TD preschool-aged children between the ages of 3 and 5, and found that accuracy with noun morphology (i.e., possessive -*s*, plural -*s*, and articles *a/the*), verb morphology (i.e., regular past tense -*ed*, third person singular -*s*, and copula and auxiliary *be*), and MLU maximized the sensitivity for discriminating between the two groups. In addition, grammatical morphology in SLI has also been explored in domains that are well-defined in typical development, such as Brown’s 14 grammatical morphemes (described in order of emergence): present progressive -*ing*, prepositions *in/on*, plural -*s*, irregular past tense, possessive -*s*, uncontractible copula, articles *a/the*, past tense -*ed*, third person singular -*s*, third person irregular, uncontractible auxiliary, contractible copula, and contractible auxiliary (Brown, 1973). While TD children master (i.e., produce 90% of the time in obligatory contexts) these morphemes in a relatively stable order between the ages of 2 and 5 (De Villiers and De Villiers, 1973), children with SLI are slower to reach mastery of correct usage of these forms in spontaneous language, and either omit them or use them incorrectly for a protracted period of time (Steckol and Leonard, 1979; Paul and Alforde, 1993). There is mixed evidence for their order of emergence in ASD (e.g., Bartolucci et al., 1980; Tek et al., 2014), so it is also unclear whether children with ASD might be slower to reach mastery of these grammatical morphemes.

Overall, these research studies in SLI show that characteristics of language impairment are identifiable from a young age, and that spontaneous language samples are a particularly useful methodology for examining group differences in grammatical abilities. As demonstrated by the studies just reviewed, frequency of overall errors as well as specific types of errors are useful in comparing children with SLI to their TD peers. Thus, we conjecture that these will also be illuminating for distinguishing amongst a heterogeneous group of children with ASD.

### Current Study

The current study examines variability in language abilities in a relatively large sample of children with ASD. Using a within-disorder approach, we highlight the characteristics of grammatical language impairment in ASD, as well as explore the potential cognitive and linguistic subgroups that exist in this sample. Using guidance from the SLI literature, spontaneous language samples will be used to categorize participants into relevant language sub-groups based on grammatical production abilities. While researchers have sub-grouped children with ASD using standardized language assessments (e.g., Kjelgaard and Tager-Flusberg, 2001; Tager-Flusberg and Joseph, 2003; Anderson et al., 2007; Rapin et al., 2009), the current study is one of the first to use language samples to more precisely capture the variability in grammatical skills in a heterogeneous sample of children with this disorder. Specifically, we will use total frequency of grammatical errors as a criterion for group membership, and propose a cut-off of 10% total grammatical errors for placing children in a grammatical language impairment subgroup. The studies that provided group means for frequency of total grammatical errors in SLI did so for children in the preschool age range, which is younger than the average age of children in the current study (*M*_age_ = 5 years, 9 months); however, based on evidence from younger TD children in these studies (Dunn et al., 1996; Eisenberg et al., 2012), we propose that 10% utterances with grammatical errors is a potentially meaningful cut-off for Kindergarten-aged children with language impairment.

Once children are classified based on total grammatical errors, we then explore group differences in the types of grammatical errors, including noun, verb, and pronoun morphology, as well as accuracy with Brown’s grammatical morphemes. These analyses address two main questions. First, is there a subgroup of 5-year-old children with ASD who have a primary impairment in grammatical language, similar to the profile of SLI, with non-verbal IQ in the normal range but frequent grammatical errors? And if so, do these children have particular difficulties with incorrect usage of verb, noun, and pronoun morphology, as well as using Brown’s 14 grammatical morphemes? Second, how does this subgroup compare to verbal children with other patterns of language and cognitive abilities?

We predict that there will be a sub-group of children who present with normal non-verbal IQ but marked difficulties with grammatical morphology. It is unclear how many children in the group will meet this criterion, as there are no documented prevalence rates for a subgroup like this in children with ASD. In addition, based on previous studies of language sub-groups in ASD, it is expected that there will also be two other language sub-groups that emerge: one, children with normal language, and two, children who also have language impairment but show a broader range of deficits, including smaller vocabularies and more atypical language. The degree to which the grammar of this latter group shows the same profile—albeit possibly in more severe form—as the grammatical impairment group is heretofore undocumented, and so will be examined for the first time in this study.

## Materials and Methods

### Participants

Participants for the current study were taken from the larger Autism Phenome Project (APP; total *N* = 189), a longitudinal project conducted at the University of California-Davis, MIND (Medical Investigation of Neurodevelopmental Disorders) Institute studying the neurobiological, genetic, and behavioral features of a large sample of children with autism. The APP recruits participants throughout northern California, with exclusionary criteria only for diagnosis, age, and language exposure (i.e., restricted to children primarily exposed to English and/or Spanish). Children were first enrolled in the APP when they were about 3-years-old (Year 1), and then a subset (*n* = 98) were seen again for behavioral testing about 2 years later when they were approximately 5-years-old (Year 3). Language abilities at Year 3 are the focus of interest for the current study, as literature on SLI suggests that grammatical language impairment can be reliably diagnosed by this age (Plante and Vance, 1994). Recordings were not available for 16 of these participants due to video recording errors (e.g., all or most of the session was not taped, or the recording file was corrupted), leaving a final sample of 82 participants for the current study.

The APP participants completed extensive behavioral testing, including some language assessments, as part of their participation in the project. This included the *Autism Diagnostic Observation Schedule* (ADOS; Lord et al., 1999), for confirmation of autism status; the *Differential Ability Scale, Second Edition* (DAS-II; Elliott, 2007), to obtain a non-verbal IQ score; and the *Peabody Picture Vocabulary Test, Third Edition* (PPVT-3; Dunn and Dunn, 1997) and *Expressive One-Word Picture Vocabulary Test, Third Edition* (EOWPVT-3; Brownell, 2000), to assess both receptive and expressive vocabulary abilities. The children were placed into three groups based on their language and non-verbal IQ testing (see **Table 1**): (1) *High Verbal* (*n* = 38) children scored in the normal range (standard scores above 85) for both non-verbal and vocabulary language testing; (2) *Low Verbal* (*n* = 11), children whose non-verbal IQ was below 85, and standardized vocabulary testing was commensurate with their non-verbal IQ; and (3) *Minimally Verbal* (*n* = 33), children whose non-verbal IQ and vocabulary performance was significantly below average (i.e., standard scores below 70).

**Table 1:** Original groups based on standardized test scores.

Measure	High verbal	Low verbal	Minimally verbal
	(*n* = 38)	(*n* = 11)	(*n* = 33)
	*M (SD)*	*M (SD)*	*M (SD)*
Age (in months)	68.63 (12.21)	66.20 (7.60)	68.88 (12.52)
NVIQ	102.95 (11.68)	78.70 (2.54)	56.29ˆa (10.22)
ADOS	11.89 (5.13)	17.80 (4.92)	22.25 (2.76)
DAS Verbal	48.44 (8.80)	32.90 (9.35)	13.81ˆb (6.52)
PPVT-3	98.47 (14.33)	75.63 (17.77)	44.67 (10.56)
EOWPVT-3	101.03 (16.52)	76.00 (12.63)	60.94 (7.86)

*NVIQ = standard score on *Differential Ability Scale, Second Edition* (DAS-II); ADOS = *Autism Diagnostic Observation Schedule*; DAS Verbal = *T*-score on DAS-II; PPVT-3 = standard score on *Peabody Picture Vocabulary Test, Third Edition*; EOWPVT-3 = standard score on *Expressive One-Word Picture Vocabulary Test, Third Edition*.*

*^a^Only 14 participants in the *Minimally Verbal* group were able to participate in the *DAS-II* testing. The remainder of this group completed the *Mullen Scale of Early Learning* (MSEL; Mullen, 1995) at Year 3, and their mean group *T*-scores on this measure was 20 (*SD* = 0), indicating floor-level performance for those children who completed the MSEL.*

*^b^This reflects the group mean for only the 14 participants in this group who participated in the DAS-II.*

Prior to transcribing the language samples, the 82 recordings were screened to identify which participants were appropriate for the project. Two participants were excluded, one who did not meet criteria for autism at Year 3 and another with significantly reduced intelligibility that was likely due to co-morbid childhood apraxia of speech. In addition, 29 children did not produce sufficient language to transcribe, which was determined based on language production during the Free Play portion of the ADOS. This was not surprising as they were all from the *Minimally Verbal* group; however, two participants from that group did produce some spontaneous language [*N* (utterances) = 33 and 124] and they were included in the final sample. This left 51 remaining participants for the language transcriptions (*M*_age_ = 68.84 months, *SD* = 12.77).

### Transcriptions

Language samples were collected from the ADOS, which provides an opportunity for investigating detailed and comprehensive grammatical profiles, as many of the tasks aim to encourage language production without providing too much structure to reduce the naturalness (Tager-Flusberg et al., 2009). Furthermore, specific tasks on the ADOS were chosen that afforded the most spontaneous and unprompted language production. Although the exact tasks varied slightly depending on the ADOS Module that the child was administered, the following tasks were included in the language sample transcriptions: Free Play, Birthday Party, Bubble Play, Snack, Make-Believe Play, Conversation, Description of a Picture, Telling a Story from a Book, Cartoons, and Creating a Story.

The language samples were transcribed by the first author and a research assistant trained in CHAT format using Computerized Language Analysis (CLAN) software (MacWhinney, 2008). Each language sample was transcribed verbatim by one of the transcribers, who viewed each recording multiple times until the entire sample was transcribed. Utterances or portions of utterances that could not be fully transcribed after three viewings were marked as unintelligible according to CHAT coding conventions. A consensus procedure to check reliability of transcripts was used, similar to that described by Shriberg et al. (1984). That is, the transcribers viewed each other’s video recordings while reading the initial transcription to check for errors or discrepancies. Discrepancies were discussed between transcribers until agreement was achieved. On the rare occasions when agreement could not be achieved, those utterances or portions of utterances were marked as unintelligible. This consensus procedure was followed for all 51 transcripts.

### Coding

CLAN conventions were deployed to perform morphological analysis on the transcripts, as well as to mark syntactic errors and extract word type and token variables for all parts of speech. Lexical measures included both types (number of unique words) and tokens (total numbers of words) for nouns, verbs, adjectives, adverbs, pronouns, and prepositions. Grammatical errors were marked within utterances to capture specific grammatical error types, including *tense-agreement errors* (omissions and usage errors for copula, auxiliary, bound tense markers, present progressive -*ing*, irregular past, and third person verb forms); *pronominal form errors* (such as person, case, and gender pronoun errors); and *noun morphology errors* (including plural -*s*, possessive -*s*, and articles *a/the*). Error counts were then converted into percentages because of the wide variability in transcript/utterance length across participants, ranging from as few as 24 utterances to as many as 247 utterances (*M* = 125.62 utterances, *SD* = 55.94). Verb tense-agreement error types were collapsed to form the Percentage of Verb Errors (PVE), calculated by dividing the number of tense-agreement errors described above by the participant’s total number of utterances. Noun morphology error types were also collapsed to form the Percentage of Noun Errors (PNE), calculated by dividing the number of noun errors described above by the total number of utterances. Finally, Percentage of Pronoun Errors (PPE) was calculated to capture the pronominal form errors as a function of the total number of utterances.

Each utterance was also coded for jargon, echolalia, and grammaticality. Echolalia is the repetition, with similar intonation, of words or phrases that someone else has said; it can be *immediate*, or right after someone said it, or *delayed*, meaning a repetition of something heard in the past (Tager-Flusberg et al., 2005). Jargon was coded if the child used strings of non-meaningful speech with odd intonation. Utterances containing echolalia or jargon were *not* coded for grammaticality. Ungrammaticality was coded if the utterance contained one or more of the grammatical marker errors described above, a word ordering error, or any other syntactic error that could not be assigned to the other categories. These errors were also calculated as proportions based on total utterances in each child’s sample, yielding variables of Percent of Echolalic Utterances (PEU), Percent of Jargon Utterances (PJU), and Percent of Ungrammatical Utterances (PUU). While we acknowledge that dialectical variation might affect our analyses, the first author, who is a trained clinician, did not observe any specific dialectical differences amongst the participants.

Finally, children’s productions of each of Brown’s 14 grammatical morphemes were examined to calculate the frequency of use, or total tokens, as well as the correct usage of those morphemes. Brown (1973) calculated accuracy in obligatory contexts as the percentage of correct usages over the total number of contexts in which an adult would be expected to use the grammatical morpheme. The procedure described by Park et al. (2012) was used in this study. Tokens were calculated by hand by examining each transcript for each occurrence of all 14 morphemes. Contexts in which each morpheme should have been used were also examined; accuracy was calculated as a percentage as total correct tokens over total obligatory contexts. For example, when a child in the sample said “and they hungry,” this was considered an omission of the contractible copula form “are” in an obligatory context, as the child should have said “and they’re hungry.” When that same child said “but they’re sad,” this was counted as a correct form of contractible copula in an obligatory context. Similarly, when another child in the sample said “it’s hat” when meaning “it’s a hat,” this was counted as an omission of the article *a* in an obligatory context; the utterance “the cat is happy” provided a correct token of the article *the* in an obligatory context. This procedure, examining correct tokens as well as omissions in contexts when an adult would have used the morpheme correctly, was repeated for all 14 of Brown’s grammatical morphemes. Once tokens as well as total obligatory contexts were calculated, correctly produced tokens over the total number of obligatory contexts was calculated, yielding a percentage of accuracy in obligatory contexts.

### Sub-grouping

The 51 verbal participants were assigned into one of three language sub-groups. The two children from the *Minimally Verbal* group who produced enough language to transcribe were combined with the children in the *Low Verbal* group to form the “Language Impaired” (LI; *n* = 13) group. The children from the *High Verbal* group were assigned into one of two groups based on their frequency of grammatical errors. Children who produced grammatical errors in more than 10% of their total utterances, as measured by PUU, were placed in the “Grammatical Impairment” group (GI; *n* = 17). Children who produced grammatical errors in fewer than 10% of their total utterances were placed in the “Language Normal” group (LN; *n* = 21).

## Results

**Table 2** presents the demographic and standardized test data for the three subgroups (LN, GI, and LI), who did not significantly differ in age, [*F*(2,48) = 0.06, *p* = 0.942]. With respect to the standardized tests, the LI Group had significantly higher ADOS scores and significantly lower non-verbal IQ, PPVT-3, and EOWPVT-3 scores than the other two groups (*p*s < 0.01). As expected given our assignment procedure, the LN and GI groups did not differ on any of these measures.

**Table 2:** Group means for standardized testing for language sample groups.

Measure	Language	Grammatical	Language	*F*-value	Interpretation
	normal (LN)	impairment (GI)	impairment (LI)		^∗∗^*p* < 0.01,
	(*n* = 21)	(*n* = 17)	(*n* = 13)		^∗∗∗^*p* < 0.001
	*M (SD)*	*M (SD)*	*M (SD)*		
Age	68.09 (12.21)	69.29 (12.56)	69.46 (14.80)	0.06	LN 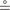 GI 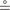 LI
NVIQ	102.38 (8.01)	103.64 (15.31)	73.53 (10.48)	31.72	LN 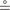 GI>LI^∗∗∗^
ADOS	11.95 (5.34)	11.82 (5.02)	18.46 (4.75)	8.06	LN 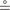 GI>LI^∗∗^
DAS Verbal	49.66 (9.12)	46.94 (8.41)	28.75 (12.87)	18.35	LN 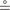 GI>LI^∗∗∗^
PPVT-3	100.90 (13.99)	95.47 (14.59)	69.60 (20.18)	14.08	LN 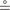 GI>LI^∗∗∗^
EOWPVT-3	104.61 (17.07)	96.59 (15.11)	73.83 (12.18)	15.53	LN 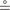 GI>LI^∗∗∗^

*Note: NVIQ = standard score on *Differential Ability Scale, Second Edition* (DAS-II); ADOS = *Autism Diagnostic Observation Schedule*; DAS Verbal = *T*-score on DAS-II; PPVT-3 = standard score on *Peabody Picture Vocabulary Test, Third Edition*; EOWPVT-3 = standard score on Expressive One-Word Picture Vocabulary Test, Third Edition*.

### Group Comparisons: Lexical Variables

**Table 3** displays the lexical variables for each group. One-way ANOVAs showed that the groups differed significantly on the number of Noun, Verb, Pronoun, Preposition, Adjective, and Adverb types and tokens they produced (*F*s > 6.0, *p*s < 0.01). *Post hoc* Tukey HSD tests revealed that the LI group produced consistently fewer types and tokens for all parts of speech when compared to both the LN and GI subgroups (*p*s < 0.01). The LN and GI subgroups did not significantly differ on any of the lexical variables.

**Table 3:** Group means for lexical measures for language sample groups.

Measure	Language	Grammatical	Language	*F*-value	Interpretation
	normal (LN)	impairment (GI)	impairment (LI)		^∗∗^*p* < 0.01,
	(*n* = 21)	(*n* = 17)	(*n* = 13)		^∗∗∗^*p* < 0.001
	*M (SD)*	*M (SD)*	*M (SD)*		
Noun types	53.62 (22.74)	52.65 (18.23)	23.08 (15.83)	11.30	LN 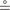 GI>LI^∗∗∗^
Noun tokens	95.14 (42.35)	102.06 (43.91)	40.77 (33.24)	9.74	LN 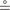 GI>LI^∗∗∗^
Verb types	40.48 (18.16)	41.06 (12.32)	19.15 (10.89)	10.37	LN 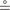 GI>LI^∗∗∗^
Verb tokens	129.62 (68.52)	143.82 (55.29)	52.92 (42.23)	10.04	LN 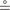 GI>LI^∗∗^
Pronoun types	18.62 (6.00)	20.88 (4.82)	10.62 (6.14)	12.99	LN 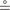 GI>LI^∗∗∗^
Pronoun tokens	102.52 (56.16)	115.29 (52.77)	39.92 (36.47)	9.08	LN 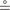 GI>LI^∗∗^
Preposition types	9.33 (5.43)	9.53 (4.29)	3.69 (3.28)	7.55	LN 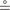 GI>LI^∗∗^
Preposition tokens	27.38 (18.64)	30.65 (19.92)	8.15 (9.67)	7.07	LN 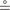 GI>LI^∗∗^
Adverb types	15.71 (9.05)	15.65 (6.36)	6.77 (4.99)	7.15	LN 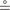 GI>LI^∗∗^
Adverb tokens	29.10 (19.12)	27.94 (13.31)	11.62 (9.31)	6.01	LN 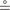 GI>LI^∗∗^
Adjective types	16.48 (8.72)	16.59 (9.84)	4.77 (3.98)	9.85	LN 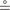 GI>LI^∗∗∗^
Adjective tokens	28.05 (14.94)	26.76 (19.68)	7.00 (5.99)	8.82	LN 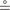 GI>LI^∗∗∗^

### Group Comparisons: Grammatical Variables

**Table 4** presents the utterance-level measures for each group. The groups differed significantly in the number of the total utterances in the sample, [*F*(2,48) = 8.896, *p* < 0.001], their mean lengths of utterance, [*F*(2,48) = 6.077, *p* < 0.01], Percentage of Ungrammatical Utterances [PUU; *F*(2,48) = 38.52, *p* < 0.001], Percentage of Echolalic Utterances [PEU; *F*(2,48) = 8.25, *p* < 0.001], and Percentage of Jargon Utterances [PJU; (*F*(2,48) = 4.33, *p* < 0.01]. Pairwise comparisons using Dunnett’s T3 test revealed that the LI group produced significantly fewer utterances than both the LN and GI subgroups (*p*s < 0.001), and that their MLUs were significantly smaller (*p*s < 0.01). The LI group also produced utterances more frequently with jargon and echolalia than the GI group (*p* < 0.05). Interestingly, while the LI subgroup produced more ungrammatical utterances than the LN group, the GI group produced significantly more ungrammatical utterances than *both* groups (*p*s < 0.001).

**Table 4:** Group means for utterance level measures for language sample groups.

Measure	Language	Grammatical	Language	Interpretation
	normal (LN)	impairment (GI)	impairment (LI)	^∗∗^*p* < 0.01,
	(*n* = 21)	(*n* = 17)	(*n* = 13)	^∗∗∗^*p* < 0.001
	*M (SD)*	*M (SD)*	*M (SD)*	
# of utterances	136.90 (44.11)	148.76 (54.30)	77.15 (48.36)	LN 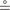 GI<LI^∗∗^
MLU	4.60 (1.71)	4.87 (1.52)	3.09 (0.84)	LN 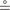 GI<LI ^∗∗^
PJU	2.66 (6.33)	1.03 (2.18)	6.87 (6.91)	LN 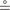 GL, GI<LI^∗^
PEU	1.77 (4.27)	0.46 (0.98)	7.63 (8.36)	LN 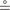 GI, GI<LI^∗^
PUU	4.05 (2.15)	15.92 (6.04)	8.81 (3.53)	LN>LI ^∗∗∗^ >GI^∗∗∗^

*Note: MLU, mean length of utterance; PJU, percentage jargon utterances; PEU, percentage echolalic utterances; PUU, percentage ungrammatical utterances.*

Next, Brown’s 14 grammatical morphemes were compared across groups. Total tokens were compared with a one-way ANOVA, revealing that the three groups differed significantly in their overall frequency of use [*F*(2,48) = 11.055, *p* < 0.001]. *Post hoc* Dunnett’s T3 comparisons confirmed that the LI group produced significantly fewer tokens of these morphemes (*M* = 38.15, *SD* = 39.13) overall compared to the LN (*M* = 119.33, *SD* = 66.09; *p* = 0.001) and GI (*M* = 124.24, *SD* = 50.71; *p* = 0.001) groups (see Appendix 1 for data and analysis by individual markers).

Correct usage in obligatory contexts of Brown’s 14 grammatical morphemes was also considered. One-way ANOVAs found significant group differences in the children’s overall percent correct usage in obligatory contexts of these morphemes [*F*(2,48) = 4.811, *p* < 0.01]. Brown (1973) considered these morphemes to be mastered when children produced them with 90% accuracy in obligatory utterances, and children in both the LN and LI subgroups reached this threshold when accuracy across all 14 morphemes was collapsed (91.7 and 92.1%, respectively). In contrast, children in the GI group performed below this threshold (81.5% accuracy). *Post hoc* Dunnett’s T3 comparisons revealed that the GI group (*M* = 81.51, *SD* = 7.69) produced significantly fewer correct uses of Brown’s morphemes in obligatory contexts than both the LN (*M* = 91.63, *SD* = 15.24; *p* = 0.034) and LI subgroup (*M* = 92.08, *SD* = 6.29; *p* = 0.001). See Appendix 2 for analyses by each individual grammatical morpheme.

The last group comparisons examined grammatical errors, as displayed in **Table 5**. One-way ANOVAs showed that the groups were significantly different in the Percentage of Noun Errors [PNE; *F*(2,48) = 7.21, *p* < 0.01], Percentage of Pronoun Errors [PPE; *F*(2,48) = 6.56, *p* < 0.01], Percentage of Verb Errors [PVE; *F*(2,48) = 17.56, *p* < 0.001], and Percentage of Overgeneralization Errors [POE; *F*(2,48) = 4.12, *p* < 0.05]. *Post hoc* Dunnett’s T3 tests revealed that the GI group made a significantly higher percentage of noun errors than the LN (*p* = 0.01) and LI groups (*p* = 0.02). In addition, they made a significantly higher percentage of pronoun errors than the LN group (*p* = 0.01). They also made a significantly higher percentage of verb tense and agreement errors than the other two groups (*p* = 0.001). Finally, the GI group (*M* = 1.2% of total utterances) made significantly more overgeneralization errors than the LI group (*M* = 0.11%), but the LN group was not significantly different than either group (*M* = 0.51%). Upon close inspection, the majority of overgeneralization errors occurred with past tense -*ed* (e.g., “won” as “winned”); and overall these overgeneralization errors were infrequent (range of 0–8 per participant across the entire sample).

**Table 5:** Group means for grammatical errors for language sample groups.

Measure	Language	Grammatical	Language	Interpretation
	normal (LN)	impairment (GI)	impairment (LI)	^∗∗^*p* < 0.01,
	(*n* = 21)	(*n* = 17)	(*n* = 13)	^∗∗∗^*p* < 0.001
	*M (SD)*	*M (SD)*	*M (SD)*	
PNE	0.14 (.65)	4.87 (1.52)	0.17 (0.44)	LN 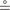 LI<GI^∗^
PPE	0.62 (1.17)	2.20 (1.72)	0.83 (1.37)	LN 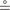 LI<GI^∗∗^
PVU	1.92 (1.51)	8.48 (5.85)	2.11 (2.19)	LN 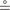 LI<GI^∗∗∗^
POE	0.51 (0.87)	1.17 (1.48)	0.11 (0.27)	LI<LN 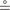 GI^∗^

*Note: PNE, percentage of noun errors; PPE, percentage pronoun errors; PVE, percentage verb errors; POE, percentage overgeneralization errors.*

## Discussion

The current study performed within-disorder comparisons of language in children with ASD using spontaneous language samples, in order to explore the wide range of linguistic ability in this population as well as to probe for the presence of different linguistic subgroups in ASD. Our findings confirm the utility of using spontaneous language samples to capture both the lexical and grammatical skills of children with autism, but more importantly, they demonstrate that there are multiple meaningful subgroups of children with ASD, which vary based on both linguistic and cognitive abilities. Specifically, we distinguished four main groups based on the language samples that were collected: first, a group of children with autism who remained minimally or non-verbal at 5 years of age and did not have enough language to produce a spontaneous sample, and second, at the other end of the spectrum, children with ASD whose standardized tests and spontaneous language samples indicated non-verbal IQ, vocabulary, and grammar at age-appropriate levels. The two ‘middle’ groups were the most interesting ones, with one subgroup of children (GI) performing in the normal range on non-verbal IQ and vocabulary testing but showing a pronounced deficit in grammatical skills in their spontaneous language, and another group of children (LI) showing deficits in non-verbal IQ, vocabulary, and grammar, but also some unexpected areas in which their speech was more similar to the LN group than the GI group.

Reviewing the findings by each group in more detail highlights the distinct features of their overall language profiles. Starting with the LN group, these children presented much more similarly to what would be expected from TD 5-year-olds. They made few grammatical morpheme errors for nouns, verbs, and pronouns; also, their accuracy with Brown’s 14 grammatical morphemes suggested mastery. Their lexical abilities also presented as intact, as they were producing a variety of word types and tokens. Thus, both grammatical and lexical abilities were judged to appropriate for their age. This group accounted for about 26.3% of our participants, similar to the rate of children in the sample from Kjelgaard and Tager-Flusberg (2001) for children with ASD who had both normal cognitive and language abilities.

Children in the LI group, who comprised 16.3% of the sample, produced much less speech than their other verbal peers with ASD; they had shorter MLUs, produced fewer tokens of grammatical morphemes, and had significantly smaller lexicons. Moreover, children in the LI group were significantly more likely to use jargon and echolalia in their utterances compared to the other two verbal groups. Atypical language like echolalia appears to be most common in children with poorer expressive language (Tager-Flusberg et al., 2005). Not surprisingly, the LI group presented with significantly lower non-verbal IQ scores than the other two groups; thus, it seems that deficits in non-verbal IQ coincide with language impairments that include smaller lexicons, more atypical language use, and less frequent grammatical marker use. Moreover, both the LI and LN groups presented with language patterns that were mostly commensurate with their non-verbal abilities and autism severity (globally low and globally high, respectively). What is possibly most interesting about the LI group, though, is that their rates of grammatical errors, including noun, verb, and pronoun errors, were comparable to the LN group, as was their accuracy with Brown’s 14 grammatical morphemes. That is, their usage of grammatical markers was not frequent, but when it occurred, was mostly correct.

This is one area where our GI subgroup, who comprised about 21.3% of the current sample, differed from the other two groups. While the GI group did not differ from the LN group on many measures from the standardized tests (non-verbal IQ, receptive and expressive vocabulary) and even some from the language samples (lexical frequency, MLU, and atypical language), grammatical errors consistently distinguished the GI group from the other two. That is, the GI group presented with significant weaknesses in their morphosyntactic production, including more frequent verb, noun, and pronoun morphology errors, as well as more overall ungrammatical language. Moreover, while the GI group was more advanced than the LI group on many measures, including manifesting higher non-verbal IQ and vocabulary testing, as well as higher MLUs, larger lexicons, and more frequent usage of grammatical markers, these two groups also differed on grammatical error rates. In fact, the language impairment of the LI group was unlike that of the GI group, in that the former’s language impairment included both low vocabulary and sparse grammatical usage but not frequent grammatical errors, while the latter group’s language impairment was specific to grammatical errors. While the exact explanations for these differences is beyond the scope of this sample, it is important to consider that there may not only be grammatical origins to these deficits, but also semantic ones. It is possible that the difficulties that the GI children have with tense markers, for example, is attributable to semantic challenges in distinguishing temporality. However, because Tovar et al. (2015) have documented that 4-year-old children with ASD successfully distinguish ongoing activities from completed actions (i.e., the ‘-ing’/past distinction) in a comprehension paradigm, we lean toward the interpretation that the challenges of the GI children in this study, for producing morphemes such as tense, are more grammatical than semantic (see also Modyanova et al., 2017). The findings are less clear for the LI group, but certainly further research that probes both semantics and grammar in the same children would be helpful in further distinguishing these two possibilities (Naigles and Tek, 2017).

Our findings align with some of the previous research that has claimed that some children with ASD meet the general criteria for SLI, evidenced by impaired grammatical skills with a relative strength in vocabulary (Kjelgaard and Tager-Flusberg, 2001; Roberts et al., 2004; Durrleman and Zufferey, 2009). In particular, there are a number of similarities between the language presentation of our GI group and that of children with SLI, such as the high rates of grammatical errors. In fact, some children in the GI group produced grammatical errors in as high as 27–28% of their utterances, a finding consistent with other studies that have explored the frequency of grammatical errors in children with SLI (e.g., Dunn et al., 1996; Eisenberg and Guo, 2013). The error types, too, specifically involving tense-verb agreement, noun markers, and pronouns, are also similar to those observed in children with SLI. Finally, the GI group produced significantly more overgeneralization errors than the LI group, and overgeneralization errors have also been found to be more common in children with SLI than children with commensurately low non-verbal IQ and language (Rice et al., 2004). The very presence of overgeneralization errors in these children with ASD is notable for another reason; namely, that this is the first documentation of overgeneralization errors for this population (cf. Eigsti et al., 2007).

These findings also lend support to theories that suggest that the acquisition of grammar depends, at least somewhat, on factors external to general cognition (e.g., Lewis and Landau, 2015; Valian, 2015; Tuller et al., 2017). That is, while this sample of children with ASD includes two subgroups whose language is generally commensurate with their non-verbal IQ (i.e., the LI and LN groups), it also includes one subgroup whose non-verbal and vocabulary abilities are high, yet whose grammatical abilities are markedly impaired. The processes and knowledge that enable the acquisition of grammar are thus shown to not be simply derived from those of general cognition; instead, they may be comprised of domain-specific configurations and computations (Rice et al., 2004; Naigles and Tek, 2017). The population of SLI has provided one clear example of this domain-specificity, as they have impaired grammar despite normal cognitive abilities (Van der Lely, 2005), and our GI group adds corroborating evidence. While the exact nature of these grammatical errors is not entirely clear and remains an issue for further investigation in ASD, our findings provide support for domain specificity of grammar in another clinical population beyond SLI.

### Limitations

There are limitations to consider about the current findings. Specifically, the classification method used in this study, categorizing children by non-verbal IQ scores and then using total number of ungrammatical utterances, was not ideal for every participant in the study. Four children in the LN group, whose non-verbal IQ was above 85 and rarely produced ungrammatical language, actually presented with considerably less language than other children in the LN group. They had much smaller MLUs and lexicons, and so based on their language, they actually may have been better suited to fall in the LI subgroup. Such ‘outlier children’ have also been attested in other studies; for example, Kjelgaard and Tager-Flusberg (2001) reported that about one-quarter of their sample did not fit neatly into language groups based on variable patterns of performance on testing. The presence of these four children in our sample raises the possibility of yet another language subgroup, one with high cognitive skills coupled with low global language; however, caution is warranted because of the small number of children who might fit this profile. And in fact, the occurrence of ‘only’ four outlier children in our study, relative to other research (Kjelgaard and Tager-Flusberg, 2001), might be taken as further support for the inclusion of detailed language samples when making such categorizations.

Another limitation was the 10% cut-off for ungrammatical utterances employed for categorizing the children with GI from the LN group. As discussed earlier, there are no normative data in typical language development for frequency of grammatical errors; therefore, there is also no universally accepted cut-off for frequency of grammatical errors in language samples for diagnosing SLI. However, based on performance between children with SLI and those who are TD (Dunn et al., 1996; Eisenberg et al., 2012), 10% was judged to be a potentially meaningful cut-off for Kindergarten-aged children, as it aligned with the grammatical rates of younger TD children in one study (Dunn et al., 1996). Certainly, this is an area important to future research so that specific delineations regarding frequency cut-offs for grammatical errors are consistent and congruent across studies.

One final limitation was our inability to describe the expressive language abilities of the *Minimally Verbal* group, as they constituted a significant proportion of children in our sample (36.3%). Although Kasari et al. (2013) recommend alternative methods like language sampling for children who are minimally verbal, the language samples from the ADOS were not an ideal measure for capturing the abilities in this sub-group. This is because most parents of children in this group reported at least some expressive vocabulary used at home. In addition, relative to parent-child and examiner-child interactions, ADOS interactions have been found to result in fewer total utterances and less complex language for children with ASD (Kover et al., 2014). While the ADOS had to be used for collecting language samples given the retrospective design of the current study, we acknowledge this may have impacted the amount of language produced by each child, particularly for those in the *Minimally Verbal* group. Unfortunately, the language sampling technique used in this study only allowed for detailed exploration of expressive grammatical abilities, and did not allow us to further explore possible receptive grammatical similarities and differences in the children with minimal language (but see Naigles and Fein, 2017).

Despite these limitations, the findings from the current study fill a critical gap in the literature that explores both language subgroups in ASD as well as the possibility of a specific grammatical impairment subgroup in this population. This is the first known study using spontaneous language samples to categorize a relatively large and heterogeneous sample of children with ASD based on both grammatical and lexical abilities. Our results suggest that verbal children with ALI diverge into two subgroups: those with a primary deficit in grammatical language but relatively intact vocabulary, and others with sparse production of both lexicon and grammar, but unexpectedly low error rates in grammatical usage as well.

### Future Directions

The current study lends support to a within-group comparison of language abilities using language samples to categorize children with ASD. Next steps with this dataset include exploring early markers of normal language and language impairment in ASD in the children at Year 1 of the APP study. Using the categorization we completed at Year 3, we will examine the language samples collected at Year 1 to discover which group differences were present earlier in development, and which predictors might be found for group membership 2 years later. In addition, APP participants included in this project had brain scans at Years 1 and 3; therefore, examination of potential neurobiological markers may also be explored as possible predictors to language group membership (e.g., Naigles et al., 2017), and as sources of information about the brain structures that underlie developing language skills in ASD.

## Ethics Statement

This study was carried out in accordance with the recommendations of the Institutional Review Board at the University of Connecticut. Because the research presented no more than minimal risk to human subjects and data was collected from video recordings made for research purposes, it qualified for expedited review and approval per Waiver of Informed Consent (45 CFR 46.116(d)). The protocol was approved by the University of Connecticut IRB.

## Author Contributions

AM, SO, and SR designed the original data collection. KW and LN worked together on the questions, design, coding, analyses, and primary write up of the current study. AM, SO, and SR provided input and final approval to this final paper.

## Conflict of Interest Statement

The authors declare that the research was conducted in the absence of any commercial or financial relationships that could be construed as a potential conflict of interest.
