# Supplementary material for: Grammatical Language Impairment in Autism Spectrum Disorder: Exploring Language Phenotypes Beyond Standardized Testing
Source: Front Psychol. 2017 Apr 18;8:532. doi: 10.3389/fpsyg.2017.00532 (PMC5394165; doi:10.3389/fpsyg.2017.00532)
Supplement: Supplementary file 1 [file Table_1.pdf]

Appendix 1 Group Means for Tokens of Brown's 14 Grammatical Morphemes for Language Sample Groups

	**Language Normal (LN)** (*n*=21)	**Grammatical Impairment (GI)** (*n*=17)	**Language Impairment (LI)** (*n*=13)	**F-value**	**Interpretation**[a,b] *p<.05, **p<.01 *** p<.001
**Morpheme**	***M (SD)***	***M (SD)***	***M (SD)***		
Present progressive -*ing*	10.52 (8.06)	10.53 (5.16)	3.62 (4.09)	5.763	LN≙GI>LI**
Preposition *in*	3.33 (3.15)	5.12 (2.96)	1.08 (1.50)	8.027	LN ≙GI> LI*
Preposition *on*	2.24 (2.00)	2.94 (3.51)	.77 (1.64)	2.753	LN ≙GI≙ LI
Plural -*s*	12.62 (8.30)	11.29 (7.89)	4.46 (3.80)	5.381	LN ≙GI> LI**
Irregular past tense	10.24 (8.22)	8.06 (6.49)	2.38 (3.62)	5.537	LN ≙GI> LI**
Possessive -*s*	.76 (.83)	.47 (.72)	.62 (.96)	0.580	LN ≙GI≙ LI
Uncontractible copula	6.57 (5.46)	7.00 (4.29)	2.08 (3.35)	4.842	LN ≙GI>LI*
Articles *a/the*	38.71 (22.11)	44.41 (23.71)	12.85 (15.47)	9.009	LN ≙GI>LI***
Past tense -*ed*	5.48 (5.29)	3.76 (3.11)	.62 (1.38)	6.183	LN≙GI>LI**
Third person singular -*s*	4.33 (4.65)	4.88 (5.11)	1.92 (2.72)	1.818	LN≙GI≙LI
Third person irregular	.76 (.83)	1.17 (1.42)	.31 (.63)	2.622	LN≙GI≙LI
Uncontractible auxiliary	2.57 (2.96)	2.24 (2.08)	.08 (.28)	5.315	LN≙GI>LI**
Contractible copula	12.95 (7.53)	13.29 (8.17)	4.31 (4.94)	7.227	LN≙GI>LI**
Contractible auxiliary	8.24 (5.99)	9.06 (6.07)	3.08 (4.29)	4.750	LN≙GI>LI*

[a]The same pattern was observed for 10 of the 14 markers individually, with the LN and GI groups producing comparable numbers of total tokens of Brown's grammatical morphemes. Group differences were not observed for the preposition *on*, the possessive -*s*, and the regular and irregular 3rd person singular.
[b]Because the assumption of homogeneity of variance was violated for some of these variables and because there were many empty cells for some participants, analyses were repeated using non-parametric statistics. Kruskal-Wallis *H* tests were thus repeated and revealed the same pattern of group effects and non-effects.

Appendix 2 Group Means for Accuracy of Brown's 14 Grammatical Morphemes in Obligatory Contexts for Language Sample Groups

	**Language Normal (LN)** (*n*=21)	**Grammatical Impairment (GI)** (*n*=17)	**Language Impairment (LI)** (*n*=13)	**F-value**	**Interpretation**[a, b] *p<.05, **p<.01 *** p<.001
**Morpheme**	***M (SD)***	***M (SD)***	***M (SD)***		
Present progressive *-ing*	91.13 (25.26)	95.26 (8.11)	100 (0)	0.971	LN≙GI≙LI
Preposition *in*	98.04 (8.08)	99.35 (2.69)	100 (0)	0.311	LN ≙GI≙ LI
Preposition *on*	93.12 (19.66)	100 (0)	90.00 (22.36)	0.919	LN ≙GI≙ LI
Plural *-s*	92.68 (18.13)	94.73 (8.67)	92.80 (15.37)	0.096	LN ≙GI≙ LI
Irregular past tense	85.95 (24.86)	62.78 (31.15)	95.64 (8.45)	5.308	LN ≙ LI, LI >GI*
Possessive *-s*	100 (0)	53.33 (50.19)	100 (0)	6.800	LN ≙ LI >GI*
Uncontractible copula	89.89 (24.37)	89.89 (24.37)	82.50 (34.54)	2.022	LN ≙GI≙ LI
Articles *a/the*	98.40 (4.42)	94.58 (6.50)	88.78 (16.39)	4.166	LN ≙GI≙ LI
Past tense *-ed*	96.54 (8.87)	70.11 (34.48)	100 (0)	5.722	LN ≙ LI >GI*
Third person singular *-s*	80.70 (34.01)	60.06 (32.66)	75.40 (36.10)	1.610	LN ≙GI≙ LI
Third person irregular	96.97 (10.04)	79.17 (39.65)	87.50 (25.00)	1.082	LN ≙GI≙ LI
Uncontractible auxiliary	100 (0)	100 (0)	100 (0)	2.483	LN ≙GI≙ LI
Contractible copula	93.49 (22.62)	93.49 (22.62)	96.87 (5.17)	0.632	LN≙GI≙LI
Contractible auxiliary	93.11 (22.50)	93.11 (22.50)	78.58 (32.99)	1.586	LN≙GI≙LI
Total Accuracy	91.63 (15.24)	81.51 (7.69)	92.08 (6.29)	4.81	HL≙LL; HL>GLI*; LL>GLI***

[a] Similar to the findings for total accuracy when all morphemes were collapsed, the GI group's accuracy was significantly lower than at least one of the other groups for the following grammatical morphemes: regular past tense, irregular past tense, and possessive -*s*. Group differences were not found for the other grammatical morphemes.

[b] Kruskal-Wallis *H* tests were repeated for accuracy in obligatory contexts, revealing nearly the same pattern of group effects and non-effects. Except the non-parametric test revealed that significant group differences were found for the uncontracted copula (H=12.70, p=.002), uncontracted auxiliary (H=6.76, p=.034), contracted copula (H=9.39, p=.009), and contracted auxiliary (H=9.112, p=.011). These group differences were not significant with the parametric statistics.
